# Delayed recognition of Ebola virus disease is associated with longer and larger outbreaks

**DOI:** 10.1080/22221751.2020.1722036

**Published:** 2020-02-04

**Authors:** M. Jeremiah Matson, Daniel S. Chertow, Vincent J. Munster

**Affiliations:** a Laboratory of Virology, Rocky Mountain Laboratories, National Institute of Allergy and Infectious Diseases, National Institutes of Health, Hamilton, MT, USA; b Marshall University Joan C. Edwards School of Medicine, Huntington, WV, USA; c Critical Care Medicine Department, Clinical Center, National Institutes of Health, Bethesda, MD, USA; d Laboratory of Immunoregulation, National Institute of Allergy and Infectious Diseases, National Institutes of Health, Bethesda, MD, USA

**Keywords:** Ebola, EBOV, Ebola virus disease, EVD, west Africa, Democratic Republic of the Congo, DRC, hemorrhagic fever

## Abstract

The average time required to detect an Ebola virus disease (EVD) outbreak following spillover of Ebola virus (EBOV) to a primary human case has remained essentially unchanged for over 40 years, with some of the longest delays in detection occurring in recent decades. In this review, our aim was to examine the relationship between delays in detection of EVD and the duration and size of outbreaks, and we report that longer delays are associated with longer and larger EVD outbreaks. Historically, EVD outbreaks have typically been comprised of less than 100 cases (median = 60) and have lasted less than 4 months (median = 118 days). The ongoing outbreak in Democratic Republic of the Congo, together with the 2013–2016 west Africa outbreak, are stark outliers amidst these trends and had two of the longest delays in detection on record. While significant progress has been made in the development of EVD countermeasures, implementation during EVD outbreaks is problematic. Thus, EVD surveillance must be improved by the broad deployment of modern diagnostic tools, as prompt recognition of EVD has the potential to stem early transmission and ultimately limit the duration and size of outbreaks.

## Introduction

**Figure 1.**
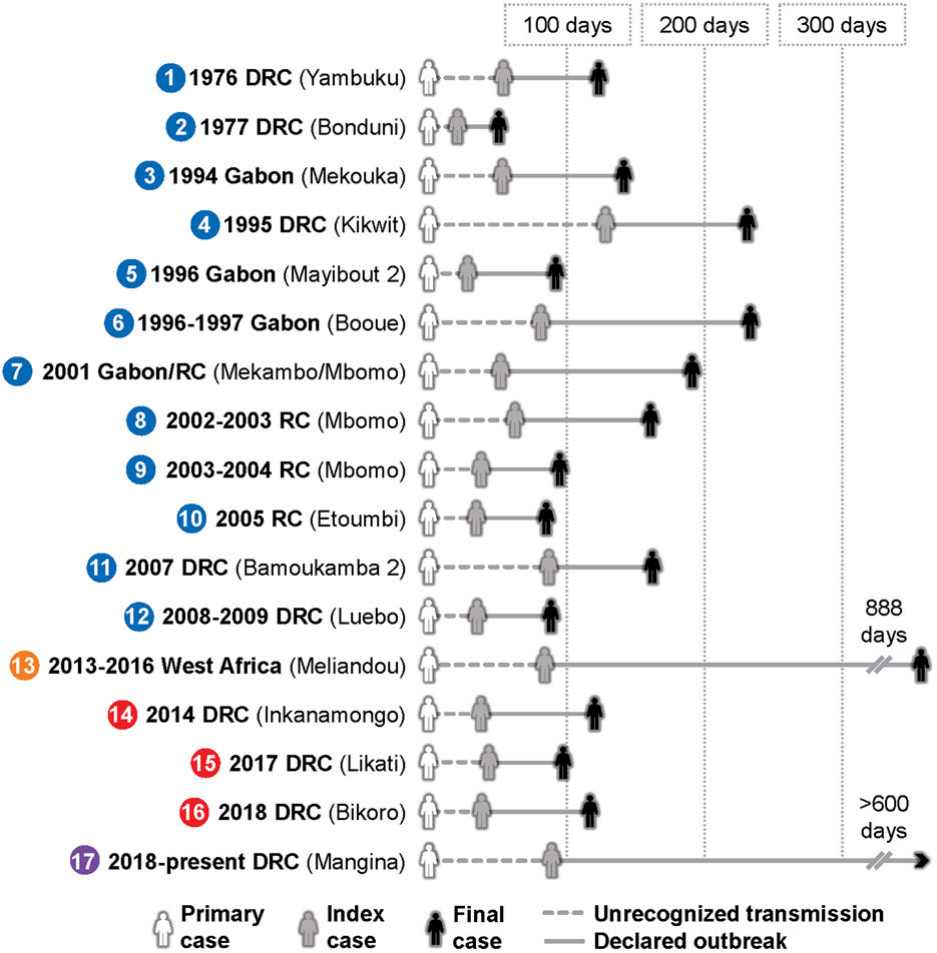
Chronology for all Ebola virus disease (EVD) outbreaks. The primary case results from zoonotic spillover and leads to a period of undetected transmission. It is typically determined retrospectively with epidemiological investigations. The index case is the first case to be recognized and marks the official beginning of an outbreak. The final case includes the 42-day observation period. Colours around numbers indicate the following groupings of outbreaks by spillover date: 1976–2012, west Africa (2013), 2014–2018, and ongoing DRC (2018). Locations in parentheses are reported outbreak spillover locations. *For the 1977 DRC (Bonduni) outbreak, the primary case, index case, and final case are the same.

Ebolaviruses are non-segmented, negative-sense, single-stranded RNA viruses in the family *Filoviridae* and the genus *Ebolavirus*. Six closely related viruses, each a member of a separate species, are currently known: Ebola virus (EBOV), species *Zaire ebolavirus*; Sudan virus (SUDV), species *Sudan ebolavirus*; Bundibugyo virus (BDBV), species *Bundibugyo ebolavirus*; Taï Forest virus (TAFV), species *Taï Forest ebolavirus*; Reston virus (RESTV), species *Reston ebolavirus*; and tentatively Bombali virus (BOMV), species *Bombali ebolavirus* [1]. Amongst these, EBOV is currently responsible for the majority of human infections and is the etiological agent of Ebola virus disease (EVD) [2]. EVD is characterized by acute onset of constitutional signs and symptoms, typically after an incubation period of 6–12 days, followed by emesis, diarrhea, multiorgan system dysfunction or failure, and occasionally hemorrhage. Fulminant cases often prove fatal within 10–14 days of symptom onset, and the EVD case fatality rate (CFR) may approach 90% [3]. For reasons which are unclear, EVD outbreaks have been occurring with increasing frequency over the past two decades in Democratic Republic of the Congo (DRC), and since mid-2018, DRC has been experiencing its largest and longest outbreak, second overall only to that which occurred in west Africa from 2013 to 2016 (Figure 1). The ongoing outbreak is the ninth of EVD in DRC; additionally, DRC previously experienced a single outbreak of Bundibugyo virus disease (BVD) in 2012, which is caused by BDBV [4].

**Figure 2.**
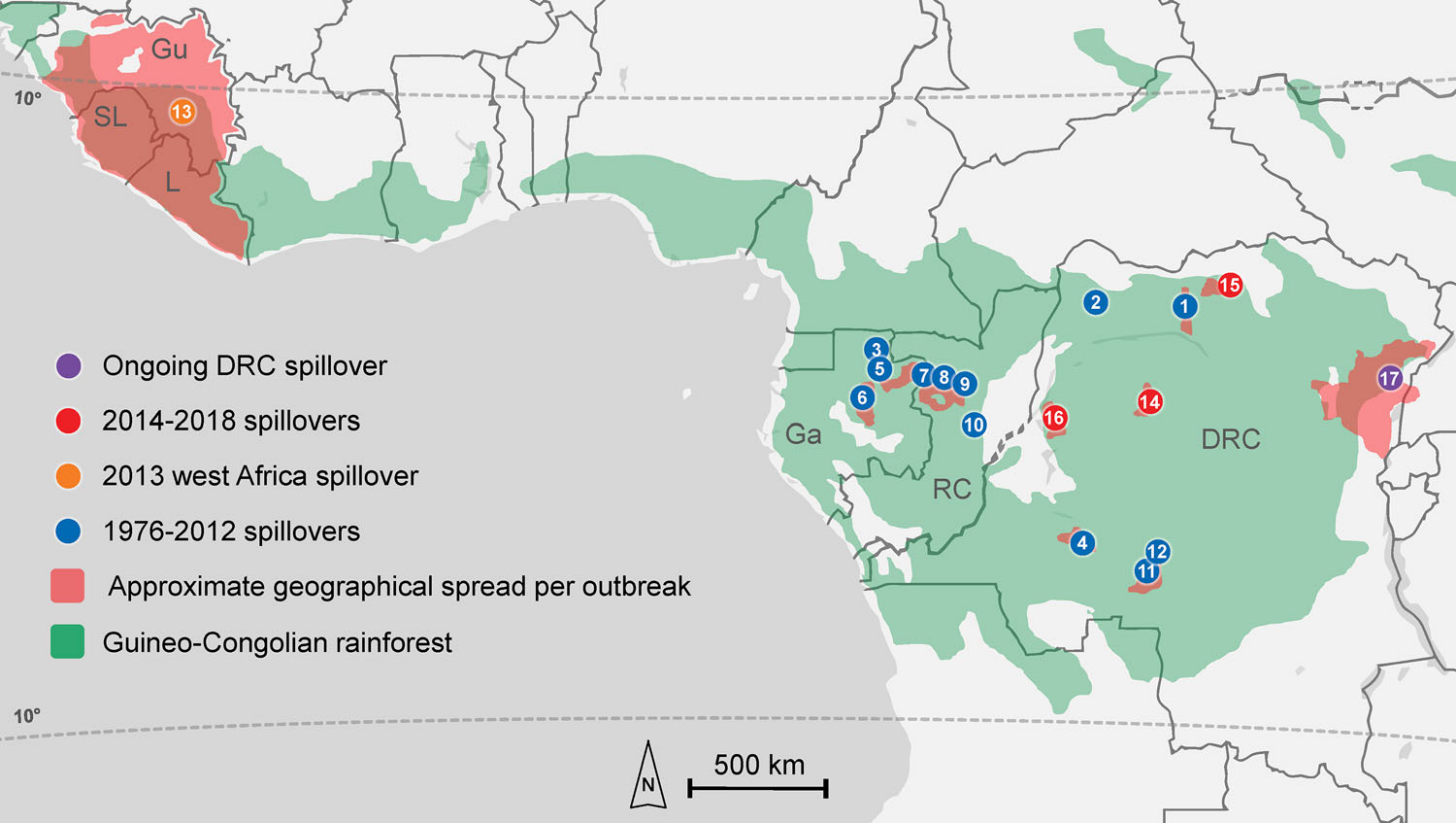
Location and spread of all currently described outbreaks of Ebola virus disease. Zoonotic spillover location per outbreak is indicated by circles with numbers. Numbers inside circles represent the order of the 17 spillovers from 1976-present correspond to Figures 1. For the 2001–2002 Gabon/Republic of Congo outbreak, which had multiple spillovers suspected, only the first spillover location is indicated. *Primary transmission zone; outbreaks 1, 6, 7, and 13 had distant case spread that is not shown in this figure; see Table 1.

EVD outbreaks are zoonotic in origin and all EBOV spillovers, including those that resulted in the west Africa and ongoing DRC outbreaks, have occurred at similar latitudes less than 10° north or south of the equator [5] and within the Guineo-Congolian rainforest terrestrial ecosystem [6] (Figure 2). Frugivorous and insectivorous bats, including *Hypsignathus monstrosus* (hammer-headed fruit bat), *Eidolon helvum* (straw-colored fruit bat), *Epomops franqueti* (Franquet's epauletted fruit bat), *Mops condylurus* (Angolan free-tailed bat), and *Miniopterus inflatus* (greater long-fingered bat), are implicated as potential natural reservoirs for EBOV [7], and other mammals including gorillas, chimpanzees, and duikers [5,8] likely act as intermediate, amplifying, dead-end hosts. Several EVD outbreaks have reported contact of the primary case with these animals, suggesting that humans may be infected by handling EBOV-infected bushmeat [9]. Despite this, EBOV has never been isolated from any naturally-infected host other than humans, hence its enzootic and epizootic transmission cycles are yet to be elucidated [5]. Furthermore, the circumstances that precipitate EBOV spillover are largely unknown and are likely a complex interplay of anthropogenic and environmental factors [10]. Although studies have reported spatiotemporal patterns in EBOV spillover [11], analyses are hampered by a relative paucity of data and the infrequent occurrence of such events. In addition, biotic and abiotic heterogeneity within the vast region of the Guineo-Congolian rainforest terrestrial ecosystem necessitates that generalizations regarding drivers of spillover be treated cautiously. Thus, definitive patterns in the ecology of EBOV that could inform public health efforts remain elusive.

However, once EBOV spillover has occurred, the dynamic and shape of most EVD outbreaks are relatively less obscure and often follow similar patterns. The aim of this review is to revisit the 17 known EVD outbreaks and examine these patterns, specifically regarding the association between the length of initial delays in detecting EVD and the subsequent duration and size of outbreaks. This association underscores the importance of early EVD detection following a spillover event and provides a strong rationale for significantly bolstering EVD diagnostic capabilities and surveillance throughout at-risk regions.

## Historical EVD outbreak patterns

**Figure 3.**
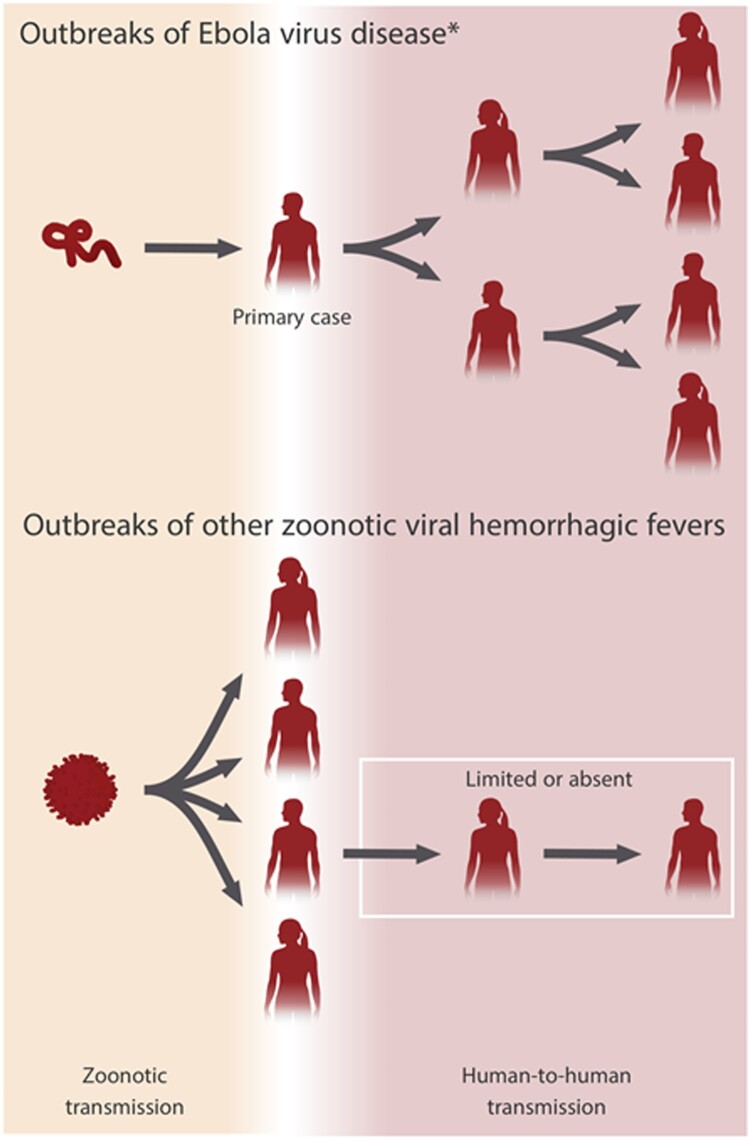
Transmission of Ebola virus (EBOV) in Ebola virus disease (EVD) outbreaks compared to virus transmission in other zoonotic viral hemorrhagic fevers. EVD outbreaks usually result from exclusively human-to-human transmission following a single zoonotic spillover to a primary EVD case. Outbreaks of other zoonotic viral hemorrhagic fevers are often characterized by sustained spillover from a reservoir/intermediate host and limited human-to-human transmission. *Other filovirus diseases (e.g. Marburg virus disease) follow a similar pattern to EVD.

Typically, as with the other filoviruses, EBOV is transmitted from an animal reservoir or intermediate host to a primary human case in a single zoonotic spillover event. The primary case subsequently initiates all human-to-human transmission. This is a unique pattern in marked contrast to outbreaks of other zoonotic viral hemorrhagic fevers (e.g. Lassa fever), which are characterized by sustained spillover and very limited human-to-human transmission (Figure 3). In EVD, once human-to-human transmission has begun it generally proceeds undetected for a period until an index case is diagnosed and recognized by health authorities, marking the official beginning and declaration of an outbreak. In most instances, the suspected primary case is then established retrospectively based on epidemiological evidence and is usually not diagnostically confirmed, thus some uncertainty is inherently present in outbreak timelines. Outbreaks continue until 42 days have elapsed after the last EVD case, which is twice the longest known EBOV incubation period of 21 days. All EVD outbreaks which began between 1976–2012 and 2014–2018 (excluding the ongoing DRC outbreak) share a generally similar dynamic and shape, while the west Africa outbreak, which began in 2013, and the ongoing DRC outbreak stand out as stark outliers.

### EVD outbreaks, 1976–2012

**Figure 4.**
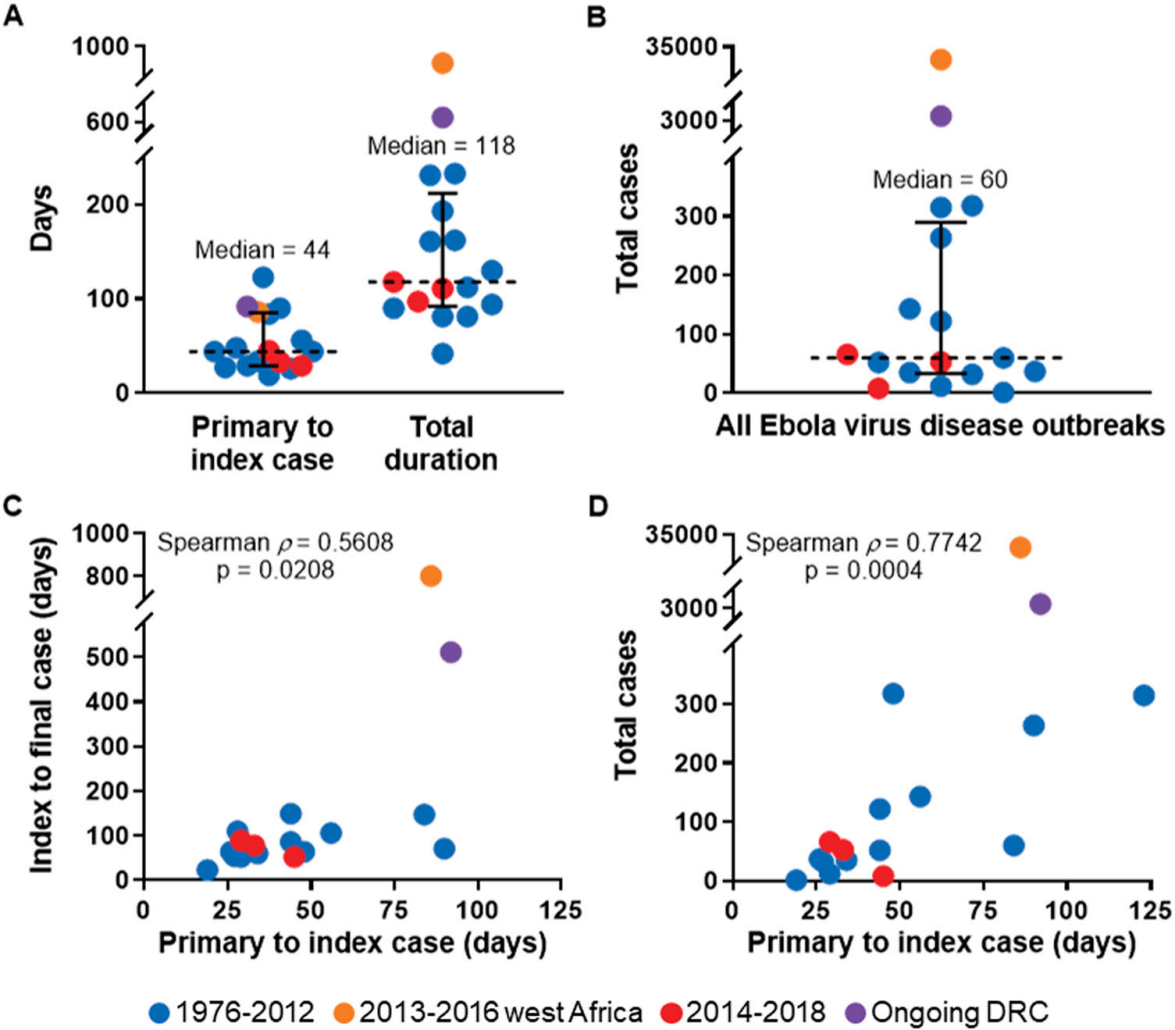
Median Ebola virus disease (EVD) outbreak metrics and correlations of outbreak duration and size to the initial period of undetected transmission. (A) Median time elapsed (dashed line) from primary case to index case and total days with interquartile range (bars) for all EVD outbreaks. (B) Median cases (dashed line) with interquartile range (bars) for all EVD outbreaks. (C) Correlation of time elapsed from suspected primary case to index case to outbreak duration for all EVD outbreaks. (D) Correlation of time elapsed from suspected primary case to index case to total cases for all EVD outbreaks.

From 1976 to 2012, 12 EVD outbreaks occurred, the largest of which was the first known occurrence of EVD in 1976 in Yambuku, DRC (318 cases). EBOV was identified as the novel etiological agent from the index patient 48 days after the symptomatic onset of the suspected primary case, and the public health measures implemented, including isolation of cases, rapid burial, and quarantine of the entire health zone proved to be effective [12]. The outbreak was declared over after 112 total days – below the median of 118 days calculated for all outbreaks (Figure 4A). In 1977, a single case of EVD was then identified in Tandala, DRC when a young girl became infected in her neighbouring village of Bonduni. Subsequently, no outbreaks were reported for nearly 20 years until EBOV reemerged in 1994 in Gabon. From 1994 to 2012, EVD outbreaks occurred with gaps of no more than a few years, and each was confined to generally remote, sparsely populated areas in Gabon [13], the Republic of Congo [8], and DRC [14]. The 1995 Kikwit, DRC outbreak was the only exception to this, with its occurrence in an urban setting amidst a substantial population (∼400,000 circa 1995) [15]. All the outbreaks during this time were ultimately successfully contained and ended with public health measures comparable to those utilized during the first outbreak in 1976.

### The west Africa EVD outbreak, 2013–2016

The west Africa outbreak marked a significant paradigm shift in the public health perception of EVD. Although some experts had maintained that EVD was a perennial threat of significant public health concern [16], it had largely come to be regarded as a minimal threat that was confined to remote populations and of very limited outbreak potential, insignificant in comparison to other infectious diseases [17,18]. Ultimately, however, approximately 30,000 cases were reported during the west Africa outbreak [19] (Figure 4B) – two orders of magnitude greater than any preceding outbreak and over 20 times the total of all previously known cases – and at 888 days in duration it was nearly four times longer than any previous outbreak (Figures 1 and 4A). EVD patients from the outbreak eventually reached 15 different countries spanning three continents as part of transmission chains or for medical treatment (Table 1), with Guinea, Liberia, and Sierra Leone at the epicenter. The World Health Organization (WHO) declared the outbreak a Public Health Emergency of International Concern (PHEIC) from 8 August 2014 until 29 March 2016, marking only the third time that such a declaration had been made. In addition to the direct public health impact of this EVD outbreak, the societal burden and economic cost were enormous, with estimates exceeding $50 billion in west Africa alone [20].

**Table 1.** EVD outbreaks with transmission and/or case(s) treated >150 km beyond primary transmission zone.

Outbreak origin	Location(s) of cases	Comments
1976 Democratic Republic of the Congo (Yambuku)	Democratic Republic of the Congo [12,21] – 3 cases: Kinshasa	Healthcare workers transported from Yambuku
1996–1997 Gabon (Booue)	Gabon [13] – 15 cases: Libreville	Patients from Booue; subsequent transmission
	South Africa [13] – 2 cases: Johannesburg	Healthcare workers from Libreville; one nosocomial case
2001–2002 Gabon/Republic of Congo (Mekambo/Mbomo)	Gabon [22] – 1 case: Franceville (transferred to Libreville for treatment)	No epidemiological links to concurrent outbreak in northern Gabon/Republic of Congo
2013–2016 Guinea (Meliandou)	Nigeria [23,24] – 20 cases: Lagos (18 cases), Port Harcourt (2 cases)	Traveller from Liberia, subsequent transmission
	United States [25–27] – 11 cases: Dallas, TX (3 cases, one transferred to Atlanta and one transferred to Bethesda for treatment)*, Atlanta, GA (3 cases + 1 transfer)*, Omaha, NE (3 cases), New York City, NY (1 case), Bethesda, MD (1 case + 1 transfer)	Seven medical evacuations; first Dallas case was a traveller from Liberia that resulted in two nosocomial transmission twice; New York City case was a healthcare worker returning from Guinea
	Mali [28] – 8 cases: Kayes (1 case), Bamako (7 cases)	Two introductions by travellers from Guinea; subsequent transmission
	United Kingdom [29,30] – 3 cases: London (2 cases + 1 transfer), Glasgow (1 case, transferred to London for treatment)	Two medical evacuations; Glasgow case was a returning healthcare worker
	Spain [31,32] – 3 cases: Madrid	Two medical evacuations; one nosocomial case
	Italy [33,34] – 2 cases: Rome	One medical evacuation; one returning healthcare worker
	Germany [35] – 3 cases: Leipzig (1 case), Hamburg (1 case), Frankfurt (1 case)	All medical evacuations
	France [36] – 2 cases: Paris	Medical evacuations
	Norway [36] – 1 case: Oslo	Medical evacuation
	Netherlands [36] – 1 case: Utrecht	Medical evacuation
	Switzerland [36] – 1 case: Geneva	Medical evacuation
	Senegal [37] – 1 case: Dakar	Traveller from Guinea

**Figure 5.**
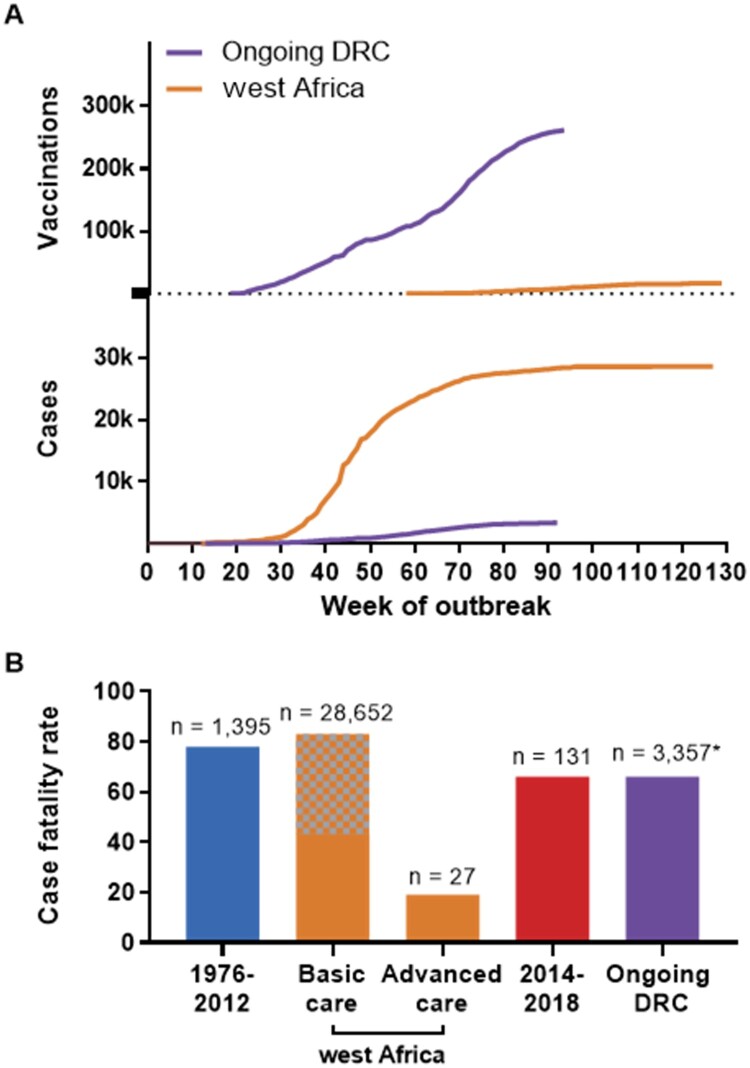
Cases and vaccinations for west Africa and the ongoing DRC Ebola virus disease outbreaks, and overall case fatality rate (CFR) for all EVD outbreaks. (A) Total cases (suspected, probable, confirmed) are included for the 2013–2016 west Africa Ebola virus disease (EVD) outbreak and the ongoing EVD outbreak in the Democratic Republic of the Congo and plotted by week. For vaccinations in west Africa, all vaccine platforms that were utilized are included; in the current DRC outbreak, Merck's V920 (rVSVΔG-ZEBOV-GP) accounts for most vaccinations; as of 24 December 2019, 2938 doses of the Johnson & Johnson Ad26.ZEBOV/MVA-BN-Filo have been administered. Beginning dates represented are 26 December 2013 and 30 April 2018 for the west Africa outbreak and the ongoing DRC outbreak, respectively. (B) Comparison of CFR for EVD cases during the following periods: 1976–2012, west Africa (2013–2016), 2014–2018, and the ongoing outbreak in the Democratic Republic of the Congo (2018-present). For west Africa, basic care is that which patients received in Ebola treatment units; advanced care is that which patients received when treated in the US or Europe. Grey and orange checkered colouring indicate that the CFR for the west Africa outbreak range from the naïve calculated 40% to recent corrected estimates of 63% [19] and 83% [44]. Numbers above bars indicate total number of cases represented. *As of 24 December 2019.

The location of the west Africa outbreak was of principal consequence: it originated 2400 km further west than any previous EVD outbreak in a region where the only warnings that filoviruses might pose a threat were a single human infection with TAFV in Côte d’Ivoire in 1994 [38] and a small outbreak of hemorrhagic fever in Pleebo, Liberia in 1995 that was suspected to be caused by an ebolavirus based on retrospective clinical and serological evidence [39]. Thus, the regional unfamiliarity with EVD in west Africa provides a rational explanation for the lengthy span of 86 days between the suspected primary case and the index case (Figure 1). Cholera was initially suspected and eventually diagnostically confirmed in a group of seven patients at a hospital in Gueckedou, although retrospectively it is likely that these patients were co-infected with EBOV based on epidemiological evidence [29]. For other early cases, a presumptive diagnosis of Lassa fever was maintained by the WHO as late as 18 March 2014 [40], less than a week before EBOV was identified. These delays in identification were key in allowing the early dissemination of EVD in southern Guinea and into the bordering regions of Sierra Leone and Liberia. Together with this undetected initial spread, numerous other factors converged to produce an exponential surge in cases (Figure 5A), including the dense and mobile population structure [41,42], delays in utilization of investigational vaccines, and community resistance to public health efforts, which sometimes turned violent [43].

### EVD outbreaks, 2014–2018

Excluding the ongoing DRC outbreak, there were three EVD outbreaks between 2014 and 2018, all largely reminiscent of those which occurred from 1976 to 2012. Each was in DRC in general proximity to areas that had experienced previous EVD outbreaks: Inkanamongo in 2014 [45], Likati in 2017 [46], and Bikoro in 2018 [47] (Figure 1). Each also adhered to historical norms for EVD outbreaks in regards to time elapsed from primary case to index case (29, 45, and 33 days, respectively) (Figures 1 and 4A), overall duration (118, 71, and 111 days, respectively) (Figures 1 and 4A), and total cases (66, 8, and 53 cases, respectively) (Figure 4B). The overarching similarity of these three outbreaks to those prior to 2013 arguably strengthened the notion that the west Africa outbreak would endure as a sole outlier.

The 2014 Inkanamongo outbreak, which took place concurrently with the west Africa outbreak, provided a particularly striking juxtaposition: it began when the west Africa outbreak had resulted in less than 2000 cases, but by the time it was declared over only four months later (Figure 1), the cases in west Africa had reached over 15,000, whereas the Inkanamongo outbreak totalled 66 cases [45]. The relatively prompt recognition of the Inkanamongo index case and the outbreak's occurrence in an area that was both comparatively remote and familiar with EVD no doubt played roles in this sharp divergence. Similarly, the 2017 Likati outbreak unfolded in an isolated area of the Bas Uele province in DRC and was one of the smallest and shortest outbreaks on record (Figure 1) thanks to timely identification of the index case, a prompt domestic response [46], and the rapid deployment of 50 WHO responders through the newly created Health Emergencies Programme, an initiative born out of the inadequacies realized in west Africa [48]. However, in April of 2018, the outbreak in Bikoro presented a significant test for EVD response efforts. It was concerning for its proximity to international borders and the potential to spread into the major urban centre of Mbandaka (population 1.2 million) [47]. Nevertheless, the outbreak was efficiently contained and ended thanks to a relatively speedy identification of the index case and decisive public health efforts. Significantly, the Bikoro outbreak also marked the first time that a vaccination campaign was utilized as an EVD outbreak countermeasure with the use of Merck's V920 vaccine (rVSVΔG-ZEBOV-GP) – a recombinant vesicular stomatitis virus vector expressing EBOV Kikwit glycoprotein (GP) that has been shown to be highly protective after a single dose, and may also be administered as a homologous prime-boost regimen [49].

### The ongoing EVD outbreak in eastern DRC, 2018-present

The ongoing DRC outbreak is thought to have begun on 30 April 2018, less than 4 weeks after the Bikoro, DRC outbreak began [50]. Despite the similar timing in their beginnings, the Bikoro outbreak came to an end one week before the index case for the ongoing eastern DRC outbreak even came to be recognized on 31 July 2018 – 92 days after the putative primary case fell ill. With this, the eastern DRC outbreak went on record with one of the longest delays in identification of the index case, despite the domestic familiarity with the disease and the tacitly heightened awareness following west Africa. It was not until a 65-year-old woman died on 25 July 2018 at Mangina referral health centre [51] and her unsafe burial resulted in seven secondary cases [52] that EVD was suspected and confirmed. This delay is perhaps associated with the spillover occurring on the outermost boundaries of the Guineo-Congolian rainforest, more than 600 km further east than in any previous EVD outbreak (Figure 2). While this region had previously experienced two outbreaks of BVD – the first in the town of Bundibugyo, Uganda in 2007 [53] and the second in the Isiro Health Zone, DRC in 2012 [4] – EVD had historically only been known much further west. Thus, the documented range of EBOV greatly expanded for the second time in only five years, following west Africa, with the easternmost and westernmost spillovers now spanning 4500 km – approximately equal in size to the continental United States. The outbreak is occurring in a densely populated urban area proximal to international borders with Rwanda, Uganda and South Sudan and major transportation thoroughfares. Distrust of public health authorities [54] and misconceptions regarding EVD [55] are proving extremely problematic given the longstanding presence of conflict and social unrest in the region. On 17 July 2019, the WHO declared the outbreak a PHEIC, making EVD responsible for two of the five times this designation has been used to date [56].

Despite these challenging circumstances, domestic spread to distant localities within DRC has not yet been reported, and international spread thus far has been limited to three cases imported to Uganda [57]. On the contrary, in addition to the broad domestic dissemination of EVD throughout Guinea, Liberia, and Sierra Leone, cases began to spread internationally during the west Africa outbreak relatively much sooner, beginning with Nigeria in July 2014 at approximately week 30 of the outbreak, and ultimately to numerous other countries (Table 1). And whereas the west Africa outbreak went on to experience a prolonged period of exponential case growth beginning around week 35, the ongoing DRC outbreak has maintained relatively slow, insidious growth (Figure 5A). While it is difficult to attribute these differences with any great degree of certainty to specific underlying factors given the complexity of the circumstances and general lack of data, the extraordinary vaccination campaign – which was promptly initiated within one week of the outbreak being recognized – in eastern DRC appears to be a major demarcating factor between the dynamics of the two outbreaks. This observation is supported by modelling analyses which suggest that EBOV vaccination significantly reduced the risk of further geographical spread of the outbreak [49]. At present, more than 250,000 doses of the rVSVΔG-ZEBOV-GP have been administered (Figure 5A) following the guidelines of the WHO Strategic Advisory Group of Experts and under expanded access to investigational new drugs [58,59]. Significantly, in a set of landmark decisions, the rVSVΔG-ZEBOV-GP received approval from the European Medicines Agency on 11 November 2019 [60] and the US Food and Drug Administration (FDA) followed suit with their own approval of the vaccine on 19 December 2019 [61], thus potentially greatly expanding access to the vaccine, principally in at-risk African nations. Furthermore, a second EVD vaccine – the Johnson & Johnson Ad26.ZEBOV/MVA-BN-Filo, a heterologous prime-boost regimen consisting of a monovalent human adenovirus serotype 26 vector expressing EBOV Mayinga GP followed by a multivalent modified vaccinia virus Ankara vector expressing EBOV Mayinga GP, Sudan virus Gulu GP, Marburg virus Musoke GP, and TAFV nucleoprotein – has been under evaluation in Uganda since August 2019 as a phase 2 clinical trial, and in November 2019 was introduced to DRC to augment use of the rVSVΔG-ZEBOV-GP vaccine in the ongoing outbreak [60,62].

### Outbreak trends summary

In summary, the median overall duration for the 17 currently described outbreaks is approximately four months (median = 118 days; interquartile range, 92–212 days) (Figure 4A). This includes the approximately one-and-a-half months (median = 44 days; interquartile range, 28–70 days) that typically elapses from the suspected time of spillover to a primary case until declaration of an outbreak following the identification of the index patient (Figure 4A). The majority of EVD outbreaks have been comprised of less than 100 human cases (median = 60; interquartile range, 34–290 cases) (Figure 4B) and have taken place in remote locations with relatively low population densities [11]. The spread of cases during most outbreaks has been limited to 150 km or less from the initial origin (Figure 2), although in a few instances cases have travelled to distant locations domestically or internationally, with further transmission sometimes occurring in those sites (Table 1).

The length of the initial period of undetected transmission between the primary case and the index case significantly correlates both with the duration (Spearman *ρ* = 0.5608, *p* = 0.0208) (Figure 4C) and total cases (Spearman *ρ* = 0.7742, *p* = 0.0004) of the ensuing outbreak (Figure 4D). As EVD outbreaks are characterized by a single zoonotic spillover followed by exclusively human-to-human transmission, this correlation is not surprising. The longer that the initial, unrecognized transmission chains propagate undetected, the longer the outbreak stands to continue due to a delay in control measures, and ultimately more infections are likely to occur. Therefore, any delay in detection of initial EVD cases following a spillover can result in uncontrolled expansion and prolonged duration of the outbreak, particularly in regions with higher population density, greater spatial connectivity, and sociopolitical unrest.

## Discussion

Significant progress has been made since the west Africa outbreak. Most notably, multiple vaccines have been developed and evaluated and are being utilized as countermeasures against EVD. Until this recent implementation of vaccination as part of the outbreak response, the public health efforts to combat EVD had remained effectively unchanged for over 40 years. Additionally, evidence suggests improved survival with basic supportive care including fluid replacement and electrolyte management [63]. The dramatically decreased CFR observed for EVD patients who received treatment in Europe and the United States [64] during the west Africa outbreak (Figure 5B) provides a strong argument for application of universal standards of care [65], and some estimate that the CFR may be less than 10% given early presentation and access to high-level intensive care [66]. Additionally, multiple promising targeted therapies [67] are currently under evaluation in a multi-arm phase III trial in DRC [68,69], with preliminary data indicating significantly improved outcomes for patients receiving either of two biologics: a single monoclonal antibody, MAb114, or a cocktail of three monoclonal antibodies, REGN-EB3 [70]. On 23 December 2019, REGN-EB3 was granted orphan drug status by the FDA, potentially increasing its accessibility for use during outbreaks [71].

Nevertheless, EVD continues to present new and sobering challenges, and the ongoing outbreak has demonstrated that containing EVD in settings with a complicated sociopolitical milieu and extensive urban infrastructure is extremely difficult. This is particularly concerning as several countries with a comparable setting, including the Central African Republic and South Sudan, are thought to be at risk for outbreaks of EVD and other filovirus diseases [72]. Perhaps the single greatest shortfall in efforts to curtail EVD outbreaks is the delay in diagnosing the index case following spillover to the primary case: three of the four longest times required for this (ongoing DRC = 92 days; 2007 Bamoukamba 2, DRC = 90 days; 2013–2016 west Africa = 86 days) have occurred approximately within the last decade (Figure 1). During the first EVD outbreak in 1976, EBOV was characterized as a novel filovirus only 48 days after the symptomatic onset of the primary case, a time approximately equal to the median for all EVD outbreaks. This required slow, labour-intensive analyses, including immunofluorescent and serological assays and electron microscopy, and the necessity for the international transportation of patient samples [12,21]. In contrast, the current gold-standard for EVD diagnostics – quantitative reverse-transcription polymerase chain reaction (qRT-PCR) – can be safely performed on location in a matter of hours with technology that has been utilized during EVD outbreaks since 1995 in Kikwit, DRC [15].

Timely recognition of an index EVD case is hampered by a lack of pathognomonic signs or symptoms at presentation, and the differential diagnosis includes malaria, typhoid fever, cholera, yellow fever, dysentery, Lassa fever, and other endemic febrile infectious diseases [12,15,21,29]. However, while prompt, accurate diagnoses are important for any disease outbreak response, the stakes for EVD are markedly higher. EVD outbreaks result from exclusively human-to-human transmission following a single zoonotic spillover, and EBOV's intrinsic capacity for relatively efficient human-to-human transmission without prior adaptation is markedly different from many other emerging viruses (Figure 3). With EVD, rapid identification and isolation of the primary case and case contacts would likely prevent further human-to-human EBOV transmission and stem the development of an EVD outbreak. Accomplishing this hinges upon incisive clinical suspicion and readily accessible diagnostics.

As even short delays in the recognition of an index EVD case may result in development of an outbreak, expansion and strengthening of basic diagnostics in western and central Africa is critical. In 2018, WHO published the first edition of its Model List of Essential In Vitro Diagnostics (EDL) as a complement to their perennial Model List of Essential Medicines [73]. The EDL recognizes the increasingly essential role that in vitro diagnostics (IVDs) have in providing accurate diagnoses and enabling public health efforts. For the primary health care level (e.g. doctor's offices, community health centres, etc.), recommended testing includes the use of rapid diagnostic tests (RDTs) utilizing capillary whole blood for endemic infectious diseases such as HIV, tuberculosis, and syphilis. More complex and confirmatory testing is recommended to then be undertaken at district hospitals and regional or national laboratories. A similar tiered diagnostic approach for EVD in countries known to be at-risk would allow for prompt detection of index EVD cases. RDTs for EVD are already available including the ReEBOV Antigen Rapid Test [74] and the OraQuick Rapid Antigen Test [75], the latter of which received crucial FDA approval on 10 October 2019 [76]. While less sensitive and/or specific than qRT-PCR based diagnostics, these antigen-based tests can provide results almost immediately at the point-of-care and should be made available at a primary health care level to enable routine screening of any suspected cases, with results confirmed with other IVDs in regional laboratories. Moreover, modern qRT-PCR platforms, such as Cepheid's cartridge-based GeneXpert, require minimal additional infrastructure and training and should be readily available for confirmation of RDT testing results. Utilization of the GeneXpert platform for EVD diagnostics is an example of a value-added approach, as a large network of these machines has been deployed through a WHO-coordinated effort for detection of multidrug-resistant TB, and is therefore also accessible for EVD diagnostics [77]. DRC began implementing GeneXperts for tuberculosis diagnostics at regional facilities in 2013, and hundreds of these instruments are already available and in use in numerous provinces across the country [78–80]. Furthermore, diagnosis of the first patients in the ongoing outbreak was accomplished with a GeneXpert, although this required shipment of the samples to the Institut National de Recherche Biomédicale in Kinshasha [81]. Given the enormous medical, economic, and political costs of EVD outbreaks, strengthening EVD diagnostic capacity to facilitate early detection of spillovers and rapid response to contain further transmission should be the highest priority.

The geographic footprint of EBOV has increased dramatically over the past five years. While EBOV spillover nevertheless remains rare, our ability to promptly detect EVD is of utmost importance. In the wake of the west Africa outbreak, nearly every state in the US established EVD diagnostics in local public health laboratories [82]. However, EVD likely only poses an indirect threat to the US; therefore, the emphasis should be focused on preparedness and outbreak prevention by bolstering front-line diagnostic capacities in the regions at direct risk of EVD outbreaks [41]. If a strong case can be made that a universal standard of care should be provided for all EVD patients [65] – which will require significant investments and capacity-building and is not preventative in nature – then certainly an equally strong or stronger case can be made for vastly increasing surveillance and diagnostic capacity for EVD throughout central Africa. Such an approach is the most efficient and effective public health strategy and should be the first-line defense in the fight against EVD.
